# A Microelectrode Array with Reproducible Performance Shows Loss of Consistency Following Functionalization with a Self-Assembled 6-Mercapto-1-hexanol Layer

**DOI:** 10.3390/s18061891

**Published:** 2018-06-09

**Authors:** Damion K. Corrigan, Vincent Vezza, Holger Schulze, Till T. Bachmann, Andrew R. Mount, Anthony J. Walton, Jonathan G. Terry

**Affiliations:** 1 EaStCHEM, School of Chemistry, The University of Edinburgh, Joseph Black Building, The King’s Buildings, West Mains Road, Edinburgh EH9 3FJ, UK; damion.corrigan@strath.ac.uk (D.K.C.); a.mount@ed.ac.uk (A.R.M.); 2 Department of Biomedical Engineering, University of Strathclyde, Glasgow G4 0NS, UK; vincent.vezza@strath.ac.uk; 3 Division of Infection and Pathway Medicine, Edinburgh Medical School, The University of Edinburgh, Chancellor’s Building, Little France Crescent, Edinburgh EH16 4SB, UK; holger.schulze@ed.ac.uk (H.S.); till.bachmann@ed.ac.uk (T.T.B.); 4 Institute for Integrated Micro and Nano Systems, School of Engineering, The University of Edinburgh, The King’s Buildings, Alexander Crum Brown Road, Edinburgh EH9 3FF, UK; anthony.walton@ed.ac.uk

**Keywords:** microfabrication, microelectrode arrays, self-assembled monolayers, electrochemical impedance spectroscopy (EIS), electrochemical sensors

## Abstract

For analytical applications involving label-free biosensors and multiple measurements, i.e., across an electrode array, it is essential to develop complete sensor systems capable of functionalization and of producing highly consistent responses. To achieve this, a multi-microelectrode device bearing twenty-four equivalent 50 µm diameter Pt disc microelectrodes was designed in an integrated 3-electrode system configuration and then fabricated. Cyclic voltammetry and electrochemical impedance spectroscopy were used for initial electrochemical characterization of the individual working electrodes. These confirmed the expected consistency of performance with a high degree of measurement reproducibility for each microelectrode across the array. With the aim of assessing the potential for production of an enhanced multi-electrode sensor for biomedical use, the working electrodes were then functionalized with 6-mercapto-1-hexanol (MCH). This is a well-known and commonly employed surface modification process, which involves the same principles of thiol attachment chemistry and self-assembled monolayer (SAM) formation commonly employed in the functionalization of electrodes and the formation of biosensors. Following this SAM formation, the reproducibility of the observed electrochemical signal between electrodes was seen to decrease markedly, compromising the ability to achieve consistent analytical measurements from the sensor array following this relatively simple and well-established surface modification. To successfully and consistently functionalize the sensors, it was necessary to dilute the constituent molecules by a factor of ten thousand to support adequate SAM formation on microelectrodes. The use of this multi-electrode device therefore demonstrates in a high throughput manner irreproducibility in the SAM formation process at the higher concentration, even though these electrodes are apparently functionalized simultaneously in the same film formation environment, confirming that the often seen significant electrode-to-electrode variation in label-free SAM biosensing films formed under such conditions is not likely to be due to variation in film deposition conditions, but rather kinetically controlled variation in the SAM layer formation process at these microelectrodes.

## 1. Introduction

Sensor systems are required for effective monitoring and control of manufacturing processes [1,2,3], measurement of water cleanliness [4,5], environmental sensing [6,7] and biomedical applications [8,9,10,11]. A range of sensing principles can be employed for system development and these include: optical, piezoelectric and electrochemical devices. It is well established that due to their high Faradaic current densities, hemispherical diffusion patterns and relative insensitivity to convection, microelectrodes offer several advantages for electrochemical sensing [12,13]. In the manufacture of microelectrode systems capable of sensing multiple analytes on a single device, photolithographic microfabrication techniques from the silicon integrated circuit industry are particularly attractive due to the ability to fabricate precise and reproducible structures of known shape and dimension [14]. Recent studies have demonstrated the successful fabrication of devices using such methods and the subsequent electrochemical measurements on electrodes of controlled shape and dimensions [15,16,17]. In addition, recent work has systematically investigated the combinations of device layers and materials to achieve optimal electrochemical responses and durability [18].

Label-free sensing is an important area, particularly for biomedical applications where fast times to result are desirable and there is a requirement to design assays requiring minimal training for operators and which do not have overly complex protocols. In addition, the need to perform multi-analyte assays is becoming increasingly important, particularly since the complexity of many modern clinical conditions has become better understood. For example, the range of antibiotic resistance genes is very large, along with the range of pathogenic organisms which can cause an infection. As a result, it is important to be able to design multiplex, label-free assays which can simultaneously detect several targets. A common approach for carrying out label-free electrochemical measurements is to use electrochemical impedance spectroscopy (EIS) [19,20] where a DC bias is imposed upon the electrode and then a small AC voltage perturbation is applied across a range of frequencies. The response, often plotted as a Nyquist plot, allows for the determination of several important physical processes taking place at the electrode-solution interface. These phenomena include the solution resistance (R_s_), the double layer capacitance (C_DL_), the charge transfer resistance (R_CT_), the Warburg impedance (W) and the non-linear resistance (R_NL_) which is usually indicative of steady state diffusion to a microelectrode. This measurement has been used for many biosensing applications and a common variant is to functionalize the electrode surface with a biological receptor (e.g., DNA or an antibody) and measure a change in R_CT_ upon binding of the target analyte [21].

Previous work has demonstrated the development of label-free biomedical assays which utilize traditionally sized “macro” electrodes and there are several well-established approaches and protocols for the functionalization and development of such electrochemical assays [22,23,24,25]. What is less commonly reported is the development of label-free measurements which utilize microelectrodes, EIS [26,27] and in particular arrays of chemically functionalized microelectrodes for biomedical measurements. This is in part due to the additional complexity of the EIS response for a microelectrode [28] and also because of the difficulties associated with reliable and reproducible manufacture of microelectrodes and effective SAM formation on microelectrode surfaces [29].

In this paper, we report the design, fabrication and characterization of a multi electrode array. Initially device performance is investigated to quantify variation between electrode sensors on the array. The electrodes are then chemically functionalized with a self-assembled monolayer (SAM) and the variation inherent following the functionalization procedure is quantified. Finally, the chemical functionalization step is modified in order to produce a more consistent and more typical microelectrode response, which points towards an enhanced method for chemically modifying microelectrode systems for label-free biosensing applications involving EIS measurements.

## 2. Materials and Methods

### 2.1. Microfabrication of Electrode Devices

**Figure 1:**
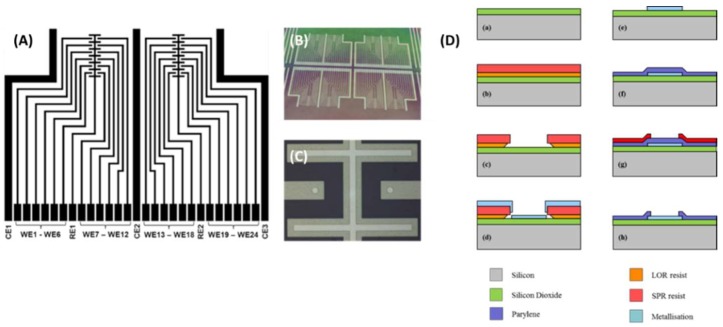
(**A**) Layout of the device. (**B**) Structures on the wafer prior to dicing. (**C**) Close up image of the working electrodes located at the end of the tracks. Reference electrode located in the spaces between the microdiscs. (**D**) Illustration of the microfabrication processes (**a**–**h**) used for device production.

The chip was designed to bear 24 equivalent 50 µm equivalent Pt disc electrodes. The electrodes are positioned in groups of 12 around two reference electrodes in a manner which means they are all equidistant from and in an equivalent configuration with respect to the relevant reference electrode (see Figure 1A,B). Each interdigitated finger has been passivated with Parylene which was later etched to expose a 50 µm disc (see Figure 1C). The chip was produced following the process flow illustrated in Figure 1D. Firstly, a 1 µm thick layer of silicon dioxide was thermally grown to provide electrical isolation between the electrodes and underlying silicon carrier wafer (a). The conductive electrode layer was patterned using a lift-off process (b,c) and the electrodes themselves comprised a 150 nm layer of platinum upon a 5 nm adhesion layer of chrome (d). To ensure that only the desired electrode areas came into contact with the solution under test, a 1 µm layer of Parylene was deposited over the samples to act as a passivation layer (f). Contact holes were then opened in the Parylene to reveal only the electrodes and contact pads of the device. This was achieved by patterning of the wafer using standard photolithography (g), followed by reactive ion etching in oxygen plasma to reveal the electrode areas (h). Full details off the fabrication process are detailed elsewhere [28].

### 2.2. Electrochemical Measurements

CV and EIS measurements were performed in a measurement buffer consisting of various concentrations of potassium ferricyanide, potassium ferrocyanide, potassium chloride and potassium nitrate. CV measurements were performed using the on chip platinum counter and Ag/AgCl/Cl^−^ reference electrode formed by placing the Ag/AgCl derivatized reference electrode in known and constant concentration Cl^−^ electrolyte solutions and the potential of the working electrode was swept between −0.05 and 0.5 V with a scan rate of 0.02 Vs^−1^. EIS measurements were performed by superimposing an AC potential of 10 mV rms onto the open circuit potential over a frequency range of 100 kHz to 0.1 Hz and measuring the current response. Thirty frequencies were measured, and frequency values were selected on a logarithmic basis. Nyquist plots were produced (Z’ vs. −Z’’) and circuit fitting was performed in order to extract values for the different circuit elements of an equivalent circuit.

### 2.3. Plating of Silver Reference Electrode and Electrode Cleaning

**Figure 2:**
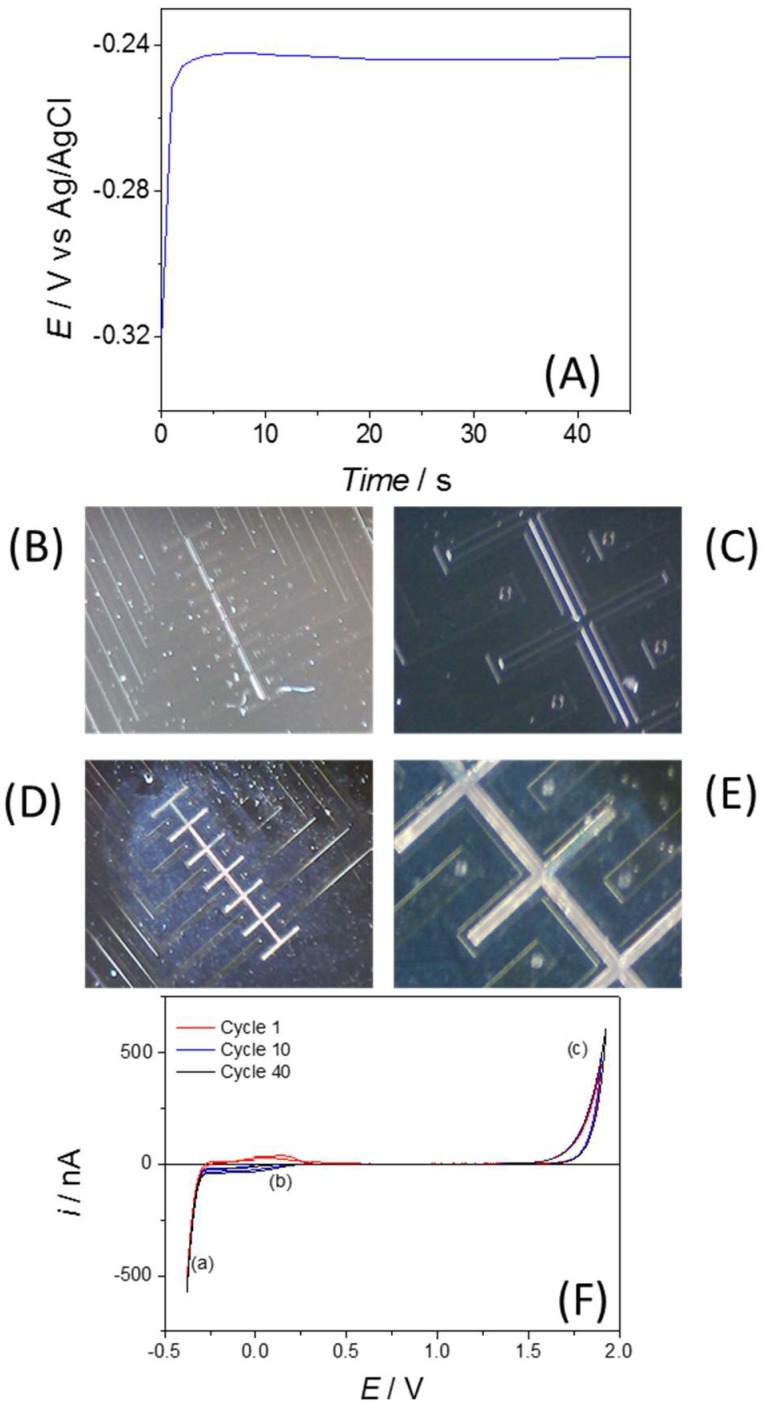
(**A**) Chronopotentiogram resulting from galvanostatic silver plating (−500 nA) of the on-chip reference electrode in 2.0 M potassium thiocyanate and 10 mM silver nitrate vs. an external Ag/AgCl/Cl^−^ (3.5 M) reference electrode. (**B**–**E**) Images of electrode devices—unplated ×20 (**B**) and ×80 (**C**) and following plating and Ag/AgCl functionalization ×20 (**D**) and ×80 (**E**). (**F**) Cleaning voltammogram of Pt microelectrodes in 0.1 M sulfuric acid.

To obtain a silver/silver chloride reference electrode it was necessary to plate silver onto the platinum structure intended for use as reference electrode. Using a three electrode system (which consists of the on-chip platinum electrode to be plated, and external Ag/AgCl reference and platinum wire counter electrodes) and a degassed solution of 2.0 M potassium thiocyanate and 20 mM silver nitrate, chronopotentiometry was performed (Figure 2A shows the chronopotentiogram). This involved passing a current of −500 µA for 45 s. Once plated, exposure of the silver surface to 50 mM ferric chloride solution for 1 min [19], resulting in the complete functionalization to Ag/AgCl. Electrode structures can be seen in Figure 2B–E respectively.

Before performing electrochemical characterization experiments, it was necessary to ensure equivalence of area and cleanliness of the microdisc electrodes. Cleaning was carried out by cycling the electrode potential between −0.35 and 1.8 V vs. an external Ag/AgCl/Cl^−^ (3 M) reference electrode in 0.1 M H_2_SO_4_ starting at 0 V, using a scan rate of 100 mVs^−1^. A cleaning voltammogram for one of the electrodes is shown in Figure 2F, in which the characteristic peaks of hydrogen absorption in the cathodic section of the scan can be seen (**a**), followed by platinum oxide reduction (**b**) and formation of platinum oxide at the anodic end of the sweep (**c**). Repeated cycling of the electrodes caused a growth in the peaks associated with cycling platinum in sulfuric acid, which eventually stabilized after 30–40 cycles. Once the voltammograms had stabilized and the electrodes showed equivalence, the cleaning process continued until all electrodes were ready for analytical measurements.

## 3. Results and Discussion

### 3.1. Electrochemical Characterisation of a 50 µM Disc from the Array

**Figure 3:**
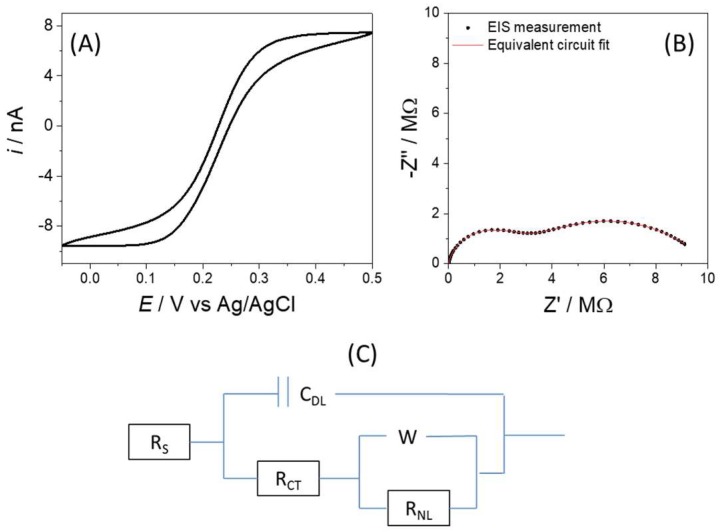
(**A**) CV and (**B**) (EIS) from a 50 µm electrode on the chip which was immersed in 1 mM potassium ferri-ferro cyanide + 1 mM potassium chloride + 100 mM potassium nitrate. Scan range—0.05 to 0.5 V and scan rate 0.02 Vs^−1^. EIS performed at open circuit potential. (**C**) Modified Randles’ equivalent circuit for EIS response of a microelectrode.

In this section, the initial electrochemical response from a single electrode is considered. The consistency of responses, which was then measured using different electrodes on the chip, is reported in later sections. Figure 3A shows the voltammetric response in 1.0 mM potassium ferri-ferrocyanide, 1.0 mM potassium chloride and 100 mM potassium nitrate solution, which is an initial characterization and for which the diffusion limiting current for the oxidation of potassium ferrocyanide to ferricyanide was evident (at and above *E* = +0.4 V) of 7.7 nA. The limiting current associated with the reduction of potassium ferricyanide to ferrocyanide (at and around *E* = 0.0 V) was larger, at −9.3 nA which is expected as a consequence of the slightly faster diffusion coefficient for this species [30]. The overall response observed in the voltammogram was typical of a microelectrode, in that a “wave”-like CV was apparent which, when the scan rate was changed, was found to be scan rate independent.

The impedimetric response was also typical of a microelectrode (Figure 3B); fitting to the established modified Randles’ equivalent circuit (Figure 3C), which includes a non-linear resistance in parallel with the Warburg impedance in order to model the hemispherical diffusion profile [28]. (EIS data is presented in the form of a Nyquist plot with each point representing a single frequency and points going from high to low frequency away from the origin). The red line in Figure 3B is the equivalent circuit fit, which produced values for *C_DL_* of 0.589 nF, *R_CT_* of 2.77 MΩ and *R_NL_* of 7.23 MΩ. The error associated with these fitted values ranged between 0.4 and 2%, confirming the goodness of fit.

Equation (1) describes the limiting current for a microdisc electrode:

*i_L_ = 4nFDc_∞_r* (1)

where *i_L_* is the limiting current, *n* is the number of electrons transferred, *F* is Faraday’s constant, *D* is the diffusion coefficient, *c_∞_* is the bulk concentration of the redox species and *r* is the electrode radius. Equation (2) describes the non-linear resistance (*R_NL_*) which is associated with the limiting current and is observed in the impedimetric response for a microelectrode:

(2) $$R_{NL}=\;\frac{4RT}{nFi_{L}}$$

Since *R_NL_* will be a more accurate measure of electrode performance due to the slight amount of double layer charging in the voltammogram, using literature values of 7.3 × 10^−6^ and 6.7 × 10^−6^ cm^2^ s^−1^ [30,31] respectively for the diffusion of ferri and ferrocyanide (298 K) to give a combined diffusion coefficient of 7.0 × 10^−6^ cm^2^ s^−1^, the fitted value for *R_NL_* and Equation (2), the total magnitude of the current is predicted to be 14.2 nA. The initial voltammetric characterization followed by EIS measurements and equivalent circuit fitting provided satisfactory evidence of microelectrode performance. Given that electrode behavior was satisfactory it was then decided to investigate inter-electrode variation across the array.

### 3.2. Electrochemical Response Following Functionalisation with 6-Mercapto-1-hexanol (MCH)

#### Cyclic Voltammetry

**Figure 4:**
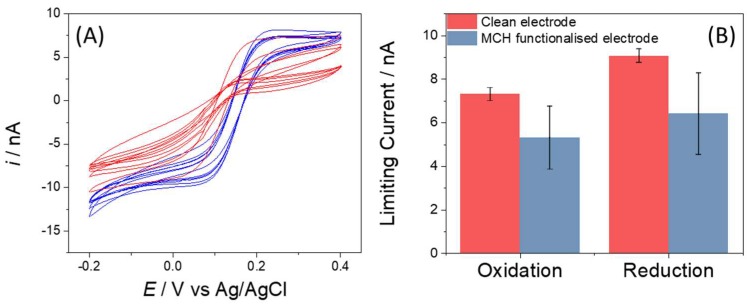
(**A**) Cyclic voltammogram from bare electrodes (blue) and following functionalization with 6 mercapto-1-hexanol (red). (**B**) EIS response from bare electrodes (blue) and following functionalization with 6 mercapto-1-hexanol (red). N = 6 and error bars represent standard deviation.

The microelectrodes were incubated with the short chain alkanethiol MCH using an established protocol [22]. The molecule forms self-assembled monolayers on platinum and gold surfaces and briefly, involved exposing the chip to a 30 µM MCH solution for 24 h with a final backfilling step of 1 h in 1.0 mM MCH solution. The formation of an alkane thiol layer is observed experimentally as an increased insulation of the electrode manifesting as a reduction in total current and an increase in impedance compared to clean electrodes. The cyclic voltammograms (Figure 4A) maintained a sigmoidal shape but showed reduced limiting currents due to a reduction in effective electrode radius due to presence of a chemical film on the surface. Figure 4B shows the mean limiting currents and standard deviations associated with the clean and functionalized electrodes. To summarize, *IL* for oxidation decreased from 7.35 to 5.31 nA with the S.D. increasing from 0.3 to 1.45 and for the reduction reaction the limiting current decreased from 9.07 nA to 6.41 nA with the S.D. increasing from 0.31 to 1.87. The decrease in limiting current is due to the formation of a blocking film, which reduces the effective area. Within the film there are pinholes which facilitate more rapid electron transfer reactions than areas bearing SAM. It is these sites which it has previously been suggested are more prevalent within SAMs formed on microelectrodes than macroelectrodes [29]. The increase in current variation as shown by increased S.D. for the limiting current reflects the irreproducibility associated with SAM formation on the electrode surface. Characterization of the electrodes as displayed in Figure 4A and reported in the S.D. associated with the limiting current showed the response to be highly consistent across the chip prior to chemical modification. These initial data recorded prior to MCH functionalization, with low inter electrode variation highlight the benefit of the microfabrication approach for microeletrode production and display similar errors to our previously published work which contrast with the higher errors typically observed from employing glass pulled microelectrodes to perform electroanalytical measurements [32].

### 3.3. Electrochemical Impedance Spectroscopy

**Figure 5:**
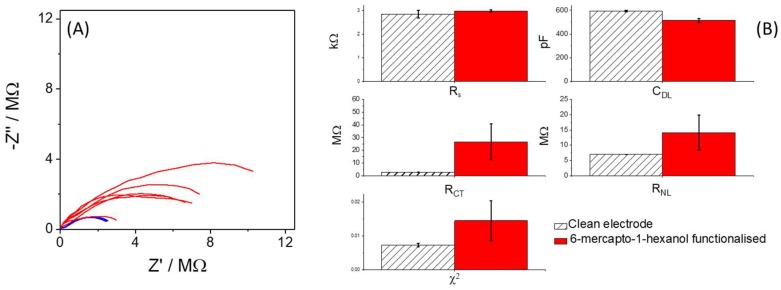
(**A**) EIS plots recorded in 2.5 mM ferri-ferrocyanide + 1 mM potassium chloride + 100 mM potassium nitrate. (**B**) Mean and Standard Deviations (Bars) associated with the parameters obtained from equivalent circuit fitting (R_S_, C_DL_, R_CT_, R_NL_ and global χ^2^) for clean and chemically modified microelectrodes (n = 6), recorded in 1.0 mM ferri-ferrocyanide + 1 mM potassium chloride + 100 mM potassium nitrate. N = 6 and error bars represent standard deviation.

All though CV experiments give an indication of electrode behavior and degree of functionalization, EIS experiments allow several important electrochemical processes to be quantified simultaneously. Following functionalization with MCH, EIS experiments revealed an overall increase in impedance (see Figure 5A,B), most obviously through increases in *R_CT_* and *R_NL_* which were indicative of blocking of the electrode surface (increased tunneling distance at sites bearing the SAM and a reduction in effective electrode radius). Figure 5A is intended as a simple visual demonstration of the increased variation in the EIS response when measured in 2.5 mM ferri-ferrocyanide before and after chemical modification. Figure 5B shows changes in the equivalent circuit parameters as fitted for EIS responses recorded in 1.0 mM potassium ferri-ferrocyanide solution and as expected, changes were observed for *C_DL_* due to the formation of a dielectric barrier, which can be modelled as a parallel plate capacitor [33]. An increase in *R_S_* was also noteworthy due to the presence of an organic film on the electrode surface changing the conductivity between working and reference electrodes. Changes in *R_S_* were not observable when using an external reference electrode to record the measurement and also not when monitoring changes in benchmark macro electrode systems during testing. This finding is interesting because it demonstrates the high sensitivity of the designed array with close spacing between WE and RE allowing visualization of a formed organic film through its presence in the conducting path. EIS data and fitted circuit parameters presented in Figure 5A,B provide a picture of increased inter electrode variation in the EIS response following SAM functionalization. Not only, as previously discussed, do the individual fitted parameters change but also the overall “*goodness of fit*” as represented by the χ^2^ value decreased following functionalization. This is visualized in Figure 5A where EIS responses from modified electrodes showed heterogeneity, raising the issue of selection of an appropriate equivalent circuit for optimum fitting of the response. To interpret these data, it is useful to consider the factors which affect the magnitude of both *R_CT_* and the limiting current (see Equations (2) and (3)).

(3) $$R_{CT}=\;\frac{4RTL}{n^{2}ADF^{2}c_{\infty }}$$

The value of the limiting currents recorded during cyclic voltammetry decreased by 28% and 29% for oxidation and reduction respectively. Changes in *I_L_* could result from a decrease in effective electrode area of a change in *D*, due to the presence of MCH film. In comparison, the fitted value of *R_CT_* increased by 740%. One way to interpret this is to consider the expressions for *I_L_*, *R_CT_* and *R_NL_* (Equations (1)–(3)). *R_CT_* is inversely proportional to the electrode area and *I_L_* and *R_NL_* proportional to and inversely proportional to the electrode radius respectively. As a result, *R_CT_* will be more sensitive to a change in effective area through formation of a film containing pinholes and this is in agreement with previously published studies which examine the formation of SAM layers on microelectrodes and conclude that increased pinhole formation is observed [29]. In this work the authors also reported that it was necessary to include additional elements in the equivalent circuit fit in order to successfully model the pinholes and defects present on a microelectrode-based SAM. However, although it should be noted that this modification to the equivalent circuit should produce better individual fits to EIS data collected on individual electrodes, it is the variation in overall impedance response across multiple electrodes in Figure 5A that is most striking, and indicates the observed variation is due to the differences in the SAM film formation process.

### 3.4. Attempts to Improve Consistency of the Chemical Modification of Sensor Surface

**Figure 6:**
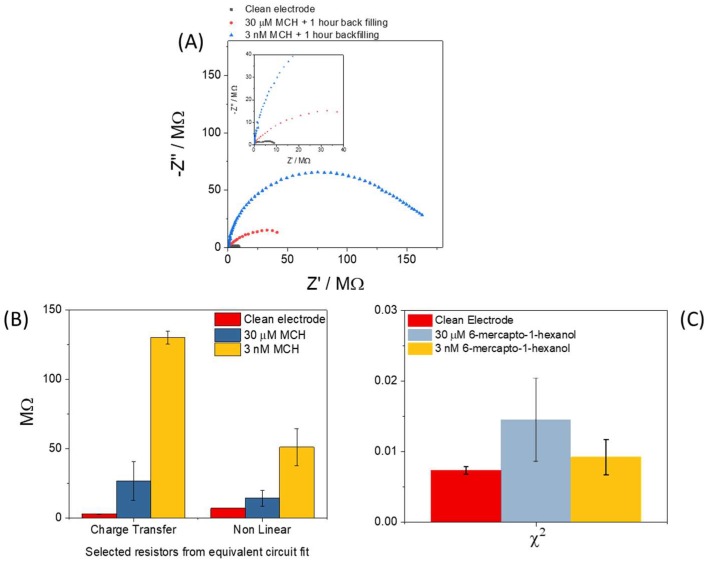
(**A**) EIS plots from a clean, 30 µM and 3 nM MCH functionalized microelectrode. (**B**) Fitted values for *R_CT_* and *R_NL_* on clean and functionalized electrodes prepared using both concentrations of MCH. (**C**) χ^2^ ‘*goodness of fit*’ values for clean and 6-mercapto-1-hexanol solutions at 30 µM and 3 nM concentrations (N = 6 and error bars represent standard deviation).

In the standard protocol developed for a macroelectrode, a sixteen hour incubation and a SAM forming solution in the micromolar concentration range, there is a large excess of available SAM forming molecules in the solution and several orders of magnitude greater timescale than that required to generate a monolayer of chemisorbed SAM molecules. For microelectrode systems with their enhanced diffusional rates this excess is even larger. Such high concentration conditions also favor the formation of multilayers, despite the weaker surface bonding characteristic of physisorption, and of SAM aggregates/micelles in solution. To produce a consistent SAM layer on microelectrodes the results presented so far show that it is necessary to modify the conditions of formation to improve reproducibility of the resulting electrochemical response. There are examples in the literature of dilution of SAM forming solutions [34] and protocols with abbreviated incubation times compared to the standard sixteen hours [26]. In this study, it was therefore decided to dilute the SAM forming molecules by a factor of ten thousand from 30 µM to 3 nM with the aim of reducing SAM aggregation and/or the degree of multilayer formation. The incubation time remained unchanged at 16 h and a 1 h backfilling step using 1.0 mM MCH was also employed. Figure 6A shows the EIS response from three electrodes: a clean electrode, an electrode incubated in 30 µM MCH plus backfilling and an electrode incubated with diluted SAM forming solution (3 nM) plus backfilling and it can be seen that the largest resistance was recorded from the electrode prepared using the diluted SAM forming solution. It can also be seen that the response appeared typical of a microelectrode with one semi-circle apparent for R_CT_ and another feature still evident at low frequencies, which is the second near semi-circle (W in parallel with R_NL_) and is indicative of steady state diffusion. Figure 6B shows the values of R_CT_ and R_NL_ with standard deviations for clean, 30 µM MCH and 3 nM MCH modified electrodes also presented. It can be seen that for both types of resistance there was a significant increase when the dilute SAM forming solution was used for chemical modification of the surface. In the case of *R_CT_*, the standard deviation reduced when the dilute SAM forming solution was employed, implying more consistent film formation. Importantly (see Figure 6C), the χ^2^ ‘*goodness of fit*’ also improved when the dilute SAM forming solution was used in contrast to the more concentrated solution. This also indicates formation of a film with greater homogeneity and fewer defects present since it is no longer necessary to include additional elements in the equivalent circuit fit to model for pinholes. This work was performed on microfabricated arrays as well as glass pulled microelectrodes in order to confirm the effect. In addition, we have previously published on the rapid formation of SAM layers using nanoelectrodes [34] and on successful formation of a DNA-based SAM for MRSA detection [26]. In both of these reported studies, increases in the magnitude of the impedance similar to the results presented herein were noted, giving additional confidence beyond the fact that six electrodes on these arrays were tested each time, that the reported R_CT_ increases and their magnitudes were realistic.

The data presented showing several responses were produced using six independent microelectrodes from across the electrode array (Figure 4, Figure 5 and Figure 6B,C). Therefore, each experiment presented has an N value of 6 and the present data set was chosen from a single experimental run in order to provide a self-consistent data set for proper analysis and comparison of circuit parameters with statistical significance. Also, in addition and over the course of the study, each experiment was repeated three times in order to confirm the reported effect of dilution of SAM molecules with a variety of redox buffer concentrations. Finally, and crucially, the immobilization experiments with 30 µM and 3 nM SAM solutions were performed using glass microelectrodes, again to confirm the reported finding. All together these data importantly highlight the variability associated with the formation of thiolated monolayers on micron scale noble metal surfaces, particularly when established functionalization protocols for macroelectrodes are employed.

## 4. Conclusions

When attempting to modify individual microelectrodes and arrays of microelectrodes for label-free biological sensing it is not advisable to use SAM forming protocols established for macroelectrodes. To take account of the improved mass transfer properties and reduced surface area of microelectrodes, by diluting the SAM forming molecule to the nanomolar range it was possible to achieve electrode functionalization with improved reproducibility and which could still be fitted using the established equivalent circuit for a microelectrode without having to include additional circuit elements to model for pinholes. These findings take account of the improved mass transfer properties and reduced surface area of microelectrodes and are useful in the development and operationalization of microelectrode sensors for biomedical applications such as the detection of DNA sequences and protein markers of disease, particularly by EIS where the reported approach allows accurate measurement of R_CT_. In addition, the presented data recorded from the microfabricated array highlight some of the benefits of this approach for electrode production, i.e., high consistency of electrochemical signal between electrodes through being able to define their critical dimensions and electrode environment accurately and reproducibly everywhere on the array, particularly when compared to data recorded using e.g., glass pulled microelectrodes. These findings point towards a higher throughput method for improving sensor functionalization and performance for biomedical sensing applications on microelectrodes, particularly where the operator is aiming to measure changes in R_CT_ based on surface hybridization or localized binding events.
